# Lateral Root Primordium Morphogenesis in Angiosperms

**DOI:** 10.3389/fpls.2019.00206

**Published:** 2019-03-19

**Authors:** Héctor H. Torres-Martínez, Gustavo Rodríguez-Alonso, Svetlana Shishkova, Joseph G. Dubrovsky

**Affiliations:** Departamento de Biología Molecular de Plantas, Instituto de Biotecnología, Universidad Nacional Autónoma de México, Cuernavaca, Mexico

**Keywords:** root development, lateral root primordium, morphogenesis, root architecture, crop species, Arabidopsis, cell proliferation, stem cells

## Abstract

Morphogenetic processes are the basis of new organ formation. Lateral roots (LRs) are the building blocks of the root system. After LR initiation and before LR emergence, a new lateral root primordium (LRP) forms. During this period, the organization and functionality of the prospective LR is defined. Thus, proper LRP morphogenesis is a decisive process during root system formation. Most current studies on LRP morphogenesis have been performed in the model species *Arabidopsis thaliana*; little is known about this process in other angiosperms. To understand LRP morphogenesis from a wider perspective, we review both contemporary and earlier studies. The latter are largely forgotten, and we attempted to integrate them into present-day research. In particular, we consider in detail the participation of parent root tissue in LRP formation, cell proliferation and timing during LRP morphogenesis, and the hormonal and genetic regulation of LRP morphogenesis. Cell type identity acquisition and new stem cell establishement during LRP morphogenesis are also considered. Within each of these facets, unanswered or poorly understood questions are identified to help define future research in the field. Finally, we discuss emerging research avenues and new technologies that could be used to answer the remaining questions in studies of LRP morphogenesis.

## Introduction

**FIGURE 1:**
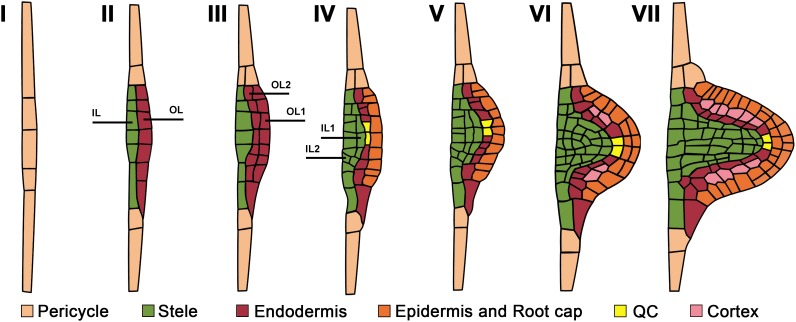
Developmental stages and cell type identity acquisition during lateral root primordium morphogenesis from initiation to lateral root emergence. Numbers correspond to the developmental stages as defined by Malamy and Benfey (1997). Emerging cell identity recognized based on cell type reporters is color-coded. Pericycle cell identity corresponds to that of the parent root. OL and IL are outer and internal layers. See text for details.

A key function of roots—water and mineral uptake and transport—is strongly related to the root system surface area. Root branching promotes and underlies the increase in root surface area, and therefore a single lateral root (LR) constitutes a building block of the root system. Thus, root branching is a ubiquitous and widely distributed process in vascular plants. A classic example of the abundance of roots is the extended root system of a single rye plant (*Secale cereale*). During only one growth season of approximately 4 months, a single plant formed 13,815,672 roots (Dittmer, 1937), most of which were LRs. LRs are initiated in the pericycle (Laskowski et al., 1995; Dubrovsky et al., 2000, 2008; Beeckman et al., 2001; Dubrovsky and Rost, 2012; Beeckman and De Smet, 2014), and comprehensive analysis of LR development has been performed on a model species, *Arabidopsis thaliana* (hereafter *Arabidopsis*). In this species, it has been recognized that LR formation is a process that includes multiple steps: (a) pericycle priming; (b) founder cell specification; (c) the first divisions in pericycle founder cells leading to LR formation, processes defined as LR initiation; (d) lateral root primordium (LRP) formation, comprising developmental processes from the first derivatives of the founder cells to formation of the dome-shaped LRP; (e) LR emergence, i.e., protrusion of the LRP through the external root tissues, including ground tissues and epidermis; (f) activation of the apical meristem in the nascent LR; and (g) LR growth (Malamy and Benfey, 1997; De Smet et al., 2003; Péret et al., 2009; Malamy, 2010; Stoeckle et al., 2018). The details of these processes are studied at different levels, from developmental anatomy to hormonal and genetic control (Casimiro et al., 2003; De Smet et al., 2006a; Fukaki and Tasaka, 2009; De Smet, 2012; Atkinson et al., 2014; Du and Scheres, 2017a; Dubrovsky and Laskowski, 2017; Ötvös and Benková, 2017). However, not all these steps are equally understood. One of the less understood steps comprises morphogenetic processes from LR initiation to LR emergence (Figure 1). Indeed, the mechanisms underlying the remarkably stable and reproducible process of formation of the three-dimensional (3D) LRP structure from a 2D plate of founder cell derivatives are a mystery. In this review, we summarize what is known about the essential elements underlying LRP morphogenesis in angiosperms and attempt to identify the basic questions related to LRP morphogenesis that remain to be answered or better understood.

## Parent Tissues Participating in Primordium Formation

Although the pericycle is a principal tissue giving rise to LRs in angiosperms, other parent root tissues, including the endodermis, cortex and vascular parenchyma, participate in LRP morphogenesis.

### Pericycle

The specification of pericycle founder cells and other pre-initiation events take place before LRP initiation (De Smet et al., 2007; Moreno-Risueno et al., 2010; Van Norman et al., 2013) and are therefore beyond the scope of this review. In *Arabidopsis*, two types of LRP initiation have been recognized: longitudinal unicellular and longitudinal bi-cellular, in which a single or two adjacent pericycle founder cells in the longitudinal plane, respectively, participate in LRP initiation (Dubrovsky et al., 2001, 2008). The most common type of initiation is considered to be longitudinal bi-cellular (Casero et al., 1995; Lloret and Casero, 2002; De Rybel et al., 2010; von Wangenheim et al., 2016). However, it cannot be excluded that the longitudinal bi-cellular type is a result of the cell division of the founder cell following the longitudinal unicellular type of initiation. Even for a model species such as *Arabidopsis*, it is not known how common each initiation type is.

**FIGURE 2:**
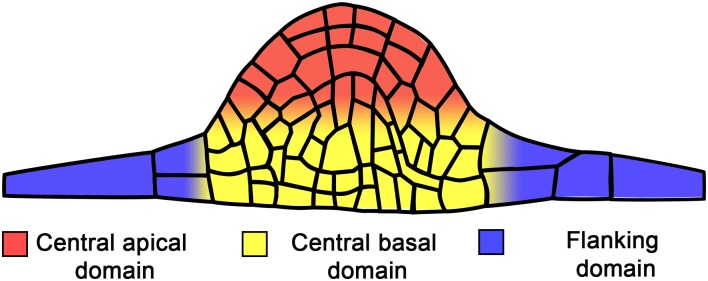
Main domains in the developing lateral root primordium.

When viewed in a transversal plane, the number of pericycle cell files that participate in the specification of the LRP founder cells varies among species; for instance, 4 to 6 phloem-adjacent files in wheat (*Triticum aestivum*; Demchenko and Demchenko, 2001) and 6–8 xylem-adjacent cell files in *Arabidopsis* (von Wangenheim et al., 2016) are involved in LRP formation. In most species, the pericycle is a unicellular tissue layer. Nevertheless, in Cucurbitaceae, two pericycle layers, internal and external, are formed in the xylem pole, and both participate in LRP formation (Dubrovsky, 1986a; Ilina et al., 2018). The most detailed analysis of pericycle participation in LRP morphogenesis has been performed in *Arabidopsis.* In this species, the first few divisions in the pericycle leading to LRP formation are anticlinal formative (asymmetrical) divisions (De Smet and Beeckman, 2011). Anticlinal divisions are perpendicular to the nearest root surface. As these divisions take place in few tangentially (i.e., in the direction perpendicular to the radius of the parent root) adjacent founder cells (Dubrovsky et al., 2001; Casimiro et al., 2003; von Wangenheim et al., 2016), a plate of on average 26 pericycle-derived cells is formed (von Wangenheim et al., 2016), corresponding to Stage (St) I, as defined by Malamy and Benfey (1997) (Figure 1). This cell plate has 2D organization and, at this point, the transition to the formation of the new growth axis that permits the 3D LRP organization is defined. The first event leading to this transition is the radial growth of StI LRP cells, resulting in the formation of the apical–basal axis of the new LR (Figure 2). This new growth direction is controlled by the adjacent endodermis through auxin signaling mediated by SHORT HYPOCOTYL2, SHY2/IAA3 (Vermeer et al., 2014). Radially expanded LRP cells eventually divide periclinally (Malamy and Benfey, 1997), i.e., parallel to the nearest root surface (Figure 1), starting in the central xylem-adjacent cell files of the plate. This division follows the established Errera’s rule, which states that cells divide preferentially along the shortest distance between cell walls (Besson and Dumais, 2011). Concurrently, the tangentially flanking cells of the plate divide in an oblique orientation, impacting the formation of the oval-shaped basal portion of the prospective LRP (Lucas et al., 2013). Starting from the two-layered LRP, 3D morphogenesis continues along the axis of the future LR. The number of cells at a given developmental stage and the division patterns vary, even though the overall LRP shape changes are conserved (Lucas et al., 2013; von Wangenheim et al., 2016). The developmental stages recognized for *Arabidopsis* (Malamy and Benfey, 1997; Napsucialy-Mendivil and Dubrovsky, 2018), depicted in Figure 1, are frequently applied to other species (Yu et al., 2016). In most angiosperms examined, pericycle participation in LRP formation is similar, at least during the early stages(Lloret and Casero, 2002).

### Endodermis and Cortex

**FIGURE 3:**
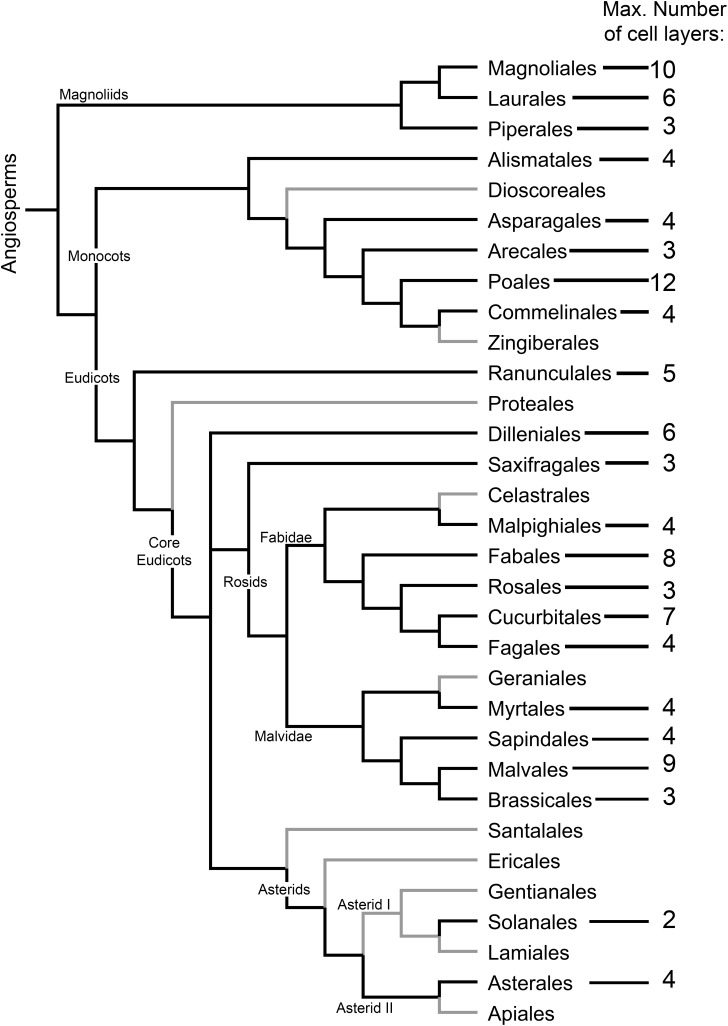
Distribution and extent of the development of the cap-like structure (CLS) of the lateral root primordium among angiosperm orders. The maximum number of CLS cell layers is indicated. Angiosperm orders for which no CLS has been found are depicted with gray branches. Cladogram topology was depicted using FigTree (http://tree.bio.ed.ac.uk/software/figtree/) following the phylogenetic relationship among angiosperms as proposed by the Angiosperm Phylogeny Group (Chase et al., 2016). The data were taken from Voronin’s (1957) analysis of Van Tieghem and Douliot (1888). Species included in the analysis were revised in accordance with contemporary taxonomic classification. Orders for which no data are available were not included in the cladogram. Additionally, data for Solanales were added (Seago, 1973; Ivanchenko et al., 2006).

As early as the 1870s, it was documented that in addition to the pericycle, other tissues participate in LRP morphogenesis (Janczewski, 1874; Van Tieghem and Douliot, 1888; Von Guttenberg, 1968). In most dicots and monocots, the endodermis is also involved in LRP formation (Van Tieghem and Douliot, 1888; Kawata and Shibayama, 1965; Bell and McCully, 1970; Seago, 1973). In some orders—for example, Poales—endodermis participation in LRP formation requires cell dedifferentiation (Danilova and Serdyuk, 1982). The first few divisions in the endodermal layer, like in the pericycle, are anticlinal (Seago, 1973; Demchenko and Demchenko, 2001). Next, in maize (*Zea mays*; Bell and McCully, 1970), *T. aestivum* (Demchenko and Demchenko, 2001), tomato (*Solanum lycopersicum*; Ivanchenko et al., 2006) and other species, the endodermal derivatives undergo periclinal divisions and form a two-layered structure. In many angiosperm taxa, even more than two layers of endodermal origin can be formed (Figure 3). The LRP tissues of endodermal origin form a temporary structure called *Tasche* in the German literature and *Poche* in the French (Clowes, 1978a). No specific term for this structure is used in the English literature. This temporary structure has some features of the root cap and sloughs off after LR emergence. Here we call this temporary structure the *Cap-Like Structure* (CLS). The CLS results from both anticlinal and periclinal divisions of the endodermis and sometimes cortex (see below). We should note here that in some cases the CLS is not temporary but a permanent structure (see below). Anatomical studies of *Z. mays* LRPs showed that the endodermis contributes to the formation of the LR’s permanent tissues, the epidermis and the root cap (Bell and McCully, 1970; Karas and McCully, 1973; McCully, 1975). This interpretation results from the fact that the epidermis of a recently emerged LR can be traced back to the endodermis of the parent root and that endodermal derivative cells in the central apical domain of the LRP start to divide periclinally and form a root cap (Bell and McCully, 1970; Karas and McCully, 1973). Particularly, when a *Z. mays* LRP protrudes about half the width of the parent root cortex, endodermal derivatives of the LRP contain abundant starch grains (Bell and McCully, 1970). By analyzing colchicine-treated chimeric LRPs that contain cells of different ploidy, Clowes (1978a) showed that the whole LR in *Z. mays* plants is of pericyclic origin and that the endodermis forms the CLS, which is maintained only for a short period after LR emergence. Similar conclusions were reached for more complex scenarios in which not only the endodermis but also the cortex participates in LRP formation (Dubrovsky, 1986a; Demchenko et al., 2001; Ilina et al., 2018), confirming the earlier view that the permanent body of the LR is entirely of pericyclic origin (Van Tieghem and Douliot, 1888).

As it is not always possible to deduce which LRP tissues are formed from the pericycle or endodermis based on anatomical observations alone, there is a need to develop cell type identity markers for this purpose. Nevertheless, anatomical studies are of great value. Based on the classical work of Philippe Van Tieghem and Douliot (1888), Voronin (1957, 1964) analyzed the types and distribution of the CLS among angiosperms. We incorporated Voronin’s data in the angiosperm phylogenetic tree proposed by the Angiosperm Phylogeny Group (Chase et al., 2016), in an effort to visualize the distribution and evolutionary trends of the appearance and types of the CLS in angiosperms (Figure 3). This analysis showed that the CLS is present in most angiosperm orders. The distribution of the CLS in angiosperms suggests an evolutionary trend toward CLS reduction until its complete disappearance, as inferred from the absence of a CLS in most orders of the recent Asterids clade (Figure 3). In *Arabidopsis*, a CLS is not found, even though it is present in other Brassicales. This ‘atypical’ pattern is perhaps a consequence of very simple root organization with a two-layered ground tissue composed of single layers of endodermis and cortex. It would be interesting to validate this hypothesis by analyzing LRP formation in *Arabidopsis* transgenic lines with supernumerary ground tissue layers that maintain endodermis identity (Nakajima et al., 2001).

**FIGURE 4:**
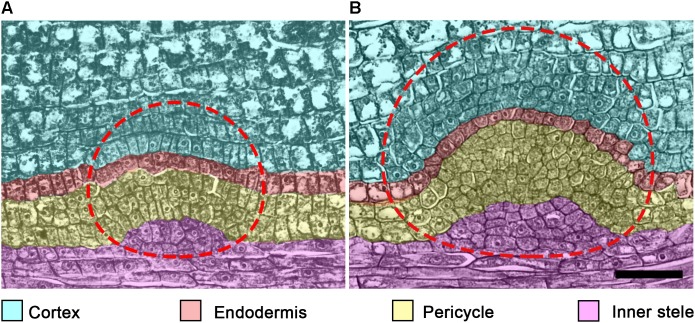
Tissues participating in lateral root primordium (LRP) formation in cucumber (*Cucumis sativus*) root. In *C. sativus*, several embryonic LRPs are formed during embryogenesis. Within the same parent root, the more apical primordium **(A)** is developed to a lesser extent than the most basal primordium **(B)**. Note the different extent of each cell type participation in LRP morphogenesis in these primordia. The temporal cap-like structure (CLS) includes endodermis and cortex derivatives of the LRP. Cell types and their derivative cells produced within the LRP are color coded. The LRP is indicated by a dashed line. Seeds were imbibed for 18 h and fixed; histological sections of the radicle were prepared and stained as described (Dubrovsky, 1986a). Scale bar = 50 μm.

In some species, the developing LRP is capable of inducing cell divisions in the adjacent cortex (Tschermak-Woess and Doležal, 1953; Demchenko et al., 2001) that are unrelated to LRP morphogenesis. The role of these divisions is unclear. In some taxa, cortex adjacent to the endodermis participates in CLS formation. This is commonly found in Fabaceae (Popham, 1955; Byrne et al., 1977) and Cucurbitaceae (Van Tieghem and Douliot, 1886; Dubrovsky, 1986a; Demchenko and Demchenko, 2001; Ilina et al., 2018), which form a massive CLS (Figure 4). Interestingly, the cortical and endodermal cells of the same files of the parent root that constitute the developing LRP participate in CLS formation and the cells outside the LRP of the same files do not divide (Dubrovsky, 1986a; Demchenko and Demchenko, 2001; Ilina et al., 2018). This endodermis continuity between the parent and lateral roots is not always maintained, and the CLS on the flanks can be destroyed before LR emergence, as in *Z. mays* (Clowes, 1978a). Few studies have examined how the ground tissue and epidermis of endodermal and cortical origin are replaced by the same cell types produced by the pericycle (Dubrovsky, 1986a).

Surprisingly, studies of CLS function during and after LRP formation are scarce. It has been suggested that CLS cells secrete hydrolases that may facilitate LR emergence (Van Tieghem and Douliot, 1888; Bonnett, 1969; McCully, 1975). The early literature on CLSs suggests that this structure protects the pericycle derivatives from mechanical damage as the LRP protrudes through the parental root tissues before LR emergence. Despite the fact that in most angiosperms the CLS is displaced by the permanent cap of pericyclic origin post-emergence, in some hydrophytes (e.g., *Hydrocharis*, *Lemna*, *Pistia*, *Eichornia*, and *Pontederia*) the CLS is permanently maintained on the LRs (Voronin, 1957).

Another possible function of the CLS might be related to cell proliferation. Cell division in the endodermis and the pericycle start simultaneously during LRP initiation and may create a critical mass of cells required to sustain rapid cell divisions (see section “The Cell Cycle During Lateral Root Primordium Morphogenesis and Timing Aspects”). Also, cell proliferation of the CLS is important for quiescent center (QC) establishment (see Section “Cell Type Identity Acquisition”). Whether endodermal participation in LRP morphogenesis in angiosperms is evolutionarily linked to the ability of this tissue in ferns to form LRPs (Mallory et al., 1970; Hou et al., 2004) is an open question.

### Vascular Parenchyma

No participation of vascular parenchyma in LRP formation has been documented in *Arabidopsis*. Therefore, this aspect of LRP morphogenesis is seldom discussed in contemporary literature. Nonetheless, the vascular parenchyma participates in primordium formation in both monocots (Rywosch, 1909; Bunning, 1952; Bell and McCully, 1970; Demchenko and Demchenko, 1996b) and eudicots (Seago, 1973; Byrne et al., 1977) by contributing to the vascular connection of the nascent LR and the parent root. It has been documented that vascular parenchyma cells start to divide very early, in StI LRPs in monocots (e.g., *T. aestivum*; Demchenko and Demchenko, 1996a) and StII LRPs in eudicots (e.g., *Glycine Max*; Byrne et al., 1977). During LRP formation in *G. max*, vascular parenchyma derivatives divide periclinally and form files of 4–5 cells that contribute to the formation of vascular tissues connecting the parent and lateral root (Byrne et al., 1977). Similarly, during embryonic LRP morphogenesis in the cucumber (*Cucumis sativus*) radicle, vascular parenchyma cells of the parent root divide 2–3 times periclinally, forming several layers of derivative cells (Dubrovsky, 1986a) (Figure 4). The contribution of vascular tissues of the parent root to LRP formation was also documented by the analysis of ploidy chimeras in *Z. mays* roots (Clowes, 1978a). Whether the participation of vascular parenchyma during LRP development is related to transport of nutrients or hormones toward the developing LRP remains to be determined.

## The Cell Cycle During Lateral Root Primordium Morphogenesis and Timing Aspects

In most angiosperms, LR initiation takes place post-germination. However, there are well-documented cases in which LRs are initiated during embryogenesis, such as in Cucurbitaceae (Clowes, 1982; Dubrovsky, 1986a,b, 1987) and Polygonaceae (buckwheat, *Fagopyrum sagittatum*, O’Dell and Foard, 1969). The extent to which LRP morphogenesis proceeds during embryogenesis ranges from StII, as in *F. sagittatum* (O’Dell and Foard, 1969), to StVII, as in *C. sativum* (Dubrovsky, 1986a). A number of embryonic LRPs are formed within the embryo (O’Dell and Foard, 1969; Dubrovsky, 1987). Interestingly, in some Cucurbitaceae in which embryonic LRP morphogenesis is documented, post-germination LRP initiation takes place in the root apical meristem (Gulyaev, 1964; Dubrovsky, 1987; Demchenko and Demchenko, 2001; Ilina et al., 2018). Some other angiosperms, especially hydrophytes from Pontederiaceae, Araceae and Alismataceae, also begin LRP morphogenesis within the apical meristem of the parent root, as reviewed elsewhere (Dubrovsky and Rost, 2003; Ilina et al., 2018). Whether there is a correlation between the species capability to start LRP morphogenesis during embryogenesis and its capacity to initiate LRPs within the root apical meristem is an open question. In most angiosperm species, however, initiation starts post-germination within the differentiation zone, where LRP morphogenesis takes place. In this review, we consider mostly these cases.

The time from LR initiation to emergence ranges from 2.8 to 3.6 days in pea (*Pisum sativum*), faba bean (*Vicia faba*), *Z. mays*, and common bean (*Phaseolus vulgaris*) (MacLeod and Thompson, 1979), is about 2.5 days in radish (*Raphanus sativus*) (Blakely et al., 1982) and 1.6–2.2 days in *Arabidopsis* (Napsucialy-Mendivil et al., 2014; von Wangenheim et al., 2016). This suggests that the whole new organ can be formed during a relatively short period. Cell cycle studies in developing LRPs using labeled DNA precursors, e.g., tritiated thymidine (^3^H-thymidine), have restrictions because LRPs at advanced stages do not incorporate ^3^H-thymidine; for example, in *V. faba* LRPs of 1,500 or fewer cells incorporated ^3^H-thymidine, whereas LRPs that contained a greater number of cells did not (Davidson and MacLeod, 1968; MacLeod and Davidson, 1968). Similarly, in monocots (*T. aestivum*), LRPs at StIII and later did not incorporate ^3^H-thymidine (Demchenko and Demchenko, 1996a). Therefore, most earlier studies were based on estimating cell doubling time (*Td*), and contemporary studies use a time-lapse approach (von Wangenheim et al., 2016). *Td* estimations assume an exponential increase in cell number in the LRP (Thompson and MacLeod, 1981) to estimate the maximal duration of the cell cycle. As all LRP cells become polyploid when treated with colchicine (MacLeod and Davidson, 1968; Friedberg and Davidson, 1971), it is accepted that all the LRP cells proliferate, and the proliferation fraction is equal to one.

Cell proliferation dynamics impact the rate of primordium formation and LRP morphogenesis. It has been proposed that the rapid establishment of an LRP after initiation might have a role in lateral inhibition—i.e., preventing the initiation of new LRPs in the vicinity of ones already initiated (Dubrovsky et al., 2001). Therefore, the cell cycle in young LRPs is expected to be shorter than that in LRPs at a later developmental stage. Indeed, a few studies show that the shortest cell cycle is found at the earliest stages of LRP morphogenesis and increases at later stages. In *V. faba*, *P. sativum*, *Z. mays*, *P. vulgaris* (MacLeod and Thompson, 1979), and *Arabidopsis* (Dubrovsky et al., 2001), the *Td* from early to later stages of LRP development increases from 8.2, 2.9, 4.5, 6.9, and 2.7 h to 14.16, 9.96, 17.65, 11.4, and 4.9 h, respectively. For *Arabidopsis*, the average *Td* in LRP cells is 7.1 h (von Wangenheim et al., 2016), about half the average cell cycle duration observed in the primary-root apical meristem (reviewed in Zhukovskaya et al., 2018). Therefore, an overall short cell cycle and a gradual increase in cell cycle duration over time seems to be a general tendency. In species with LRPs already initiated during embryogenesis, the opposite trend is found post-germination. For instance, in *C. sativus*, *Td* is the longest (8.7 h) when pre-initiated LRP cells first enter the cell cycle soon after seed imbibition and decreases to 2.7 h in the LRP just before LR emergence (Dubrovsky, 1986b), explaining why LRs emerge rapidly after germination in this species.

When *V. faba* LRPs are about to emerge, their cells are less proliferatively active than during previous stages; after emergence, a sharp increase in proliferation is observed (Friedberg and Davidson, 1971; MacLeod, 1972, 1973). The rate of formation of individual LRPs within a parent root is variable, as documented for *V. faba*, *P. sativum*, *Z. mays*, and *P. vulgaris* (MacLeod and Thompson, 1979) and *Arabidopsis* (Dubrovsky et al., 2006; von Wangenheim et al., 2016). This is in agreement with the fact that, contrary to LR initiation, LRP formation along the parent root does not follow an acropetal pattern and is asynchronous. The heterogeneity in the rate of LRP formation explains why younger LRPs are found among the older ones or among emerged LRs, even in the zone where the vascular cambium and secondary tissues are formed (Napsucialy-Mendivil and Dubrovsky, 2018). Whether slow developing or delayed LRPs are capable of later resuming development is not well documented and remains an open question.

The processes of LR initiation and emergence are coordinated (Lucas et al., 2008b). The distance from the apex of the parent root to the site where the LR emerges depends on the site of LR initiation and on the rate of primordium formation. As mentioned above, in *Arabidopsis*, the time between LRP initiation and LR emergence is one of the shortest reported in Angiosperms. Nonetheless, the LR emerges a few centimeters from the apex. When LRs are initiated in the root apical meristem of the parent roots, as in Cucurbitaceae, LRs emerge at a shorter distance, e.g., 12–15 mm from the primary root apex in squash, *Cucurbita pepo* (Demchenko and Demchenko, 2001).

The role of cell cycle duration in LRP morphogenesis has not been extensively studied. The central domains of the LRP seem to develop faster than the flanking domains. Figure 2 shows the terminology used to describe the LRP domains. The progeny of central founder cells is characterized by an average interphase duration of 6.0 h, whereas the corresponding period in the progeny of peripheral founder cells is 7.2 h (von Wangenheim et al., 2016). This suggests that the difference between cell cycle duration in each cell lineage has a profound impact on morphogenesis. It is not understood how heterogeneity in cell cycle time is related to LRP morphogenesis, whether the differences in cell cycle duration in certain domains have a critical role in defining the dome shape of the developing LRP, or whether cell cycle time heterogeneity dictates the shape of the LRP or if the shape defines the cell cycle duration in different domains. Future studies should address these questions.

## Hormonal Regulation of Morphogenesis

Since early studies, the importance of hormonal regulation in all aspects of LR development was recognized (Wightman et al., 1980). The role of auxin in LR development is well documented (Fukaki and Tasaka, 2009; Lavenus et al., 2013; Du and Scheres, 2017a) and LRP morphogenesis is known to depend on endogenous auxins up to StIV (Laskowski et al., 1995). In *P. sativum* plants treated with auxin transport inhibitors, normal LRP dome organization is lost and the primordium structure is transformed into a globular mass of cells (Hinchee and Rost, 1992), highlighting the significance of auxin in LRP morphogenesis. Accordingly, synthetic auxin-response promoter *DIRECT REPEAT5 (DR5*) (Ulmasov et al., 1997) activity is high during both LR initiation (Dubrovsky et al., 2008) and throughout LRP development (Benková et al., 2003; Dubrovsky et al., 2008). The auxin response maximum is present starting from StI in LRPs. From StIII, it is restricted to the central apical domain (Figure 2) of the LRP, corresponding to the prospective location of the QC (Dubrovsky et al., 2008). The auxin maximum apparently has a role in stem cell niche establishment and thus is important for normal LRP morphogenesis (see also the next section).

The localized auxin maximum response is present in different angiosperm orders, from monocots (*Z. mays*, Jansen et al., 2012) to eudicots (*S. lycopersicum* and *Arabidopsis*, Dubrovsky et al., 2008). It has been shown that while LR initiation does not depend on shoot-derived auxin, this source of auxin is essential for post-initiation LRP morphogenesis and LR emergence (Bhalerao et al., 2002; Ditengou et al., 2008; Swarup et al., 2008; Richter et al., 2009). A recent study strongly suggests that formation of an auxin maximum in the LRP also depends on local auxin synthesis in proliferating cells (Brumos et al., 2018). In most angiosperms, LR initiation starts in the young differentiation zone. Interestingly, when the LRP forms within the root apical meristem of the parent root, as in *C. pepo*, the auxin maximum is also established from the very early stages of LRP formation and is subsequently maintained throughout LRP development (Ilina et al., 2018). This suggests that LRP morphogenesis depends on auxin regardless of the differentiation state of the parent root cells giving rise to the LRP.

The auxin gradient with a maximum in the central apical domain (Figure 2) of the developing LRP is created by the auxin efflux carriers PIN-FORMED 1 (PIN1), PIN3, PIN4, PIN6, and PIN7 (Benková et al., 2003; Marhavý et al., 2014) and the auxin influx carriers AUX1 and LIKE AUX 3 (LAX3) (Marchant et al., 2002; Péret et al., 2012b). This gradient is formed by auxin flux from the flanking to the central domain and from the basal to the apical domain (Figure 2). Mutations that affect auxin transport lead to abnormal LRP formation. In the *pin1* mutant, LRP formation is slow and DR5 promoter activity is extended to more cells (Benková et al., 2003). Similarly, LRP formation takes more time in a *lax3* mutant (Swarup et al., 2008). Additionally, similar phenotypes are found in single and multiple mutants in *LATERAL ORGAN BOUNDARIES-DOMAIN/ASYMMETRIC LEAVES2-LIKE* (*LBD*) genes encoding transcription factors, such as *lbd29* (Porco et al., 2016), *lbd16 lbd18* double and *lbd16 labd18 lbd29* triple mutants (Feng et al., 2012; Lee et al., 2015), which are targets of auxin signaling through AUXIN RESPONSE FACTOR 7 (ARF7) and ARF19 transcription factors (Okushima et al., 2007). These phenotypes are explained by the finding that LBDs directly or indirectly promote expression of auxin influx carriers (Feng et al., 2012; Lee et al., 2015; Porco et al., 2016).

In the p*in1 pin3 pin4* triple mutant, treatment with exogenous auxin results in a massive division of pericycle cells and formation of a multilayered pericycle without a defined LRP structure, a phenotype similar to that of wild-type (Wt) seedlings treated with auxin transport inhibitor (Benková et al., 2003). Similarly, the role of auxin in correct cell division orientation during LRP morphogenesis is shown in *pin2 pin3 pin7* triple mutants that form fused LRs (Laskowski et al., 2008). *S. lycopersicum DIAGEOTROPICA (DGT)* encodes Cyclophilin A, which negatively regulates PIN protein localization and thereby affects both LRP initiation and morphogenesis (Ivanchenko et al., 2015). When pericycle cell proliferation is induced by auxin treatment in a *dgt* mutant, massive cell proliferation without formation of a recognizable primordium is observed (Ivanchenko et al., 2006). Auxin response restriction to the central apical domain of the LRP depends on the APETALA2-class transcription factor *PLETHORA* (PLT) genes (Du and Scheres, 2017b), whose expression in turn depends on AUXIN RESPONSE FACTOR 7 (ARF7) and ARF19 (Hofhuis et al., 2013). In the *plt3 plt5 plt7* triple mutant, DR5 activity is more diffuse and PIN1 and PIN3 expression is low or absent. In addition, due to abolishment of the auxin gradient in the mutant, periclinal cell divisions in LRPs are delayed or absent from StII onwards (Du and Scheres, 2017b). This work underlines the importance of auxin in LRP morphogenesis and cell division orientation. In line with this, it was shown that the correct orientation of pericycle cell divisions leading to LRP formation is abolished when the adjacent endodermal cell is ablated, but it is restored when exogenous auxin is added (Marhavý et al., 2016).

The processes of LRP initiation and LRP morphogenesis are linked, as the first cell division of founder cells triggers a new developmental program that permits *de novo* organ formation (Dubrovsky et al., 2008). This triggering is dependent on ARF7, ARF19, and INDOLE-3 ACETIC ACID 14 (IAA14)/SOLITARY-ROOT, the latter of which represses ARFs (Fukaki et al., 2005). Importantly, in the lateral rootless mutant *solitary root* (*slr*)*/iaa14* (Fukaki et al., 2002) and double mutant *arf7 arf19* (Okushima et al., 2005; Wilmoth et al., 2005), LRP initiation is almost completely abolished, although some StI but no StII LRPs still form. This phenotype cannot be rescued by the application of exogenous auxin (Fukaki et al., 2002; Okushima et al., 2005; Wilmoth et al., 2005).

**FIGURE 5:**
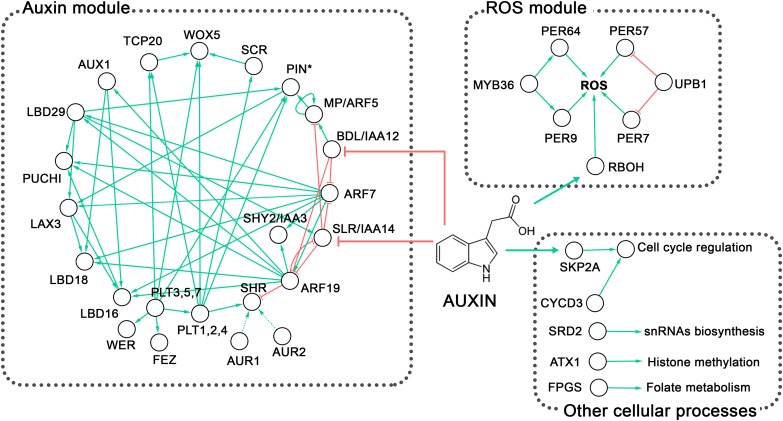
Overview of the genetic control of lateral root primordium (LRP) morphogenesis in *Arabidopsis*. Two well-defined regulatory modules are observed, the auxin regulatory and the reactive oxygen species (ROS) homeostasis modules (discussed in the text). The first module involving ARFs, IAAs, AUX/LAX and PIN proteins (PIN^∗^) also includes the PLT, PUCHI, FEZ, and WER transcription factors; the GRAS transcription cofactors SCR and SHR; and LBD proteins. The Ser/Thr kinases AUR1 and AUR2 are highly redundant and AlphaScreen assays showed that AUR1 interacts with SHR (Takagi et al., 2016); however, this interaction has not been proven *in planta* and hence is depicted with a discontinuous line. The second module includes the transcription factors MYB36 and UPB1, which control the expression of *PER* genes. Peroxidases together with RBOH control ROS homeostasis. Other cellular processes mentioned in the text and involved in LRP formation, such as the cell cycle, snRNA biosynthesis, histone methylation, and folate metabolism, are also depicted. Although particular genes involved in the regulation of these processes have been shown to affect LRP morphogenesis, their interactions with larger gene regulatory modules await to be discovered. Green arrows indicate activation or positive interaction; red lines with blunt ends indicate downregulation. The network was built from literature mining and visualized in Cytoscape v3.2.1 (Shannon et al., 2003). Complete names of the proteins are provided in the text.

Overexpression of *CYCLIND3;1* in the *slr* mutant background promotes cell proliferation in pericycle cells but no organized LRPs are formed beyond StI (Vanneste et al., 2005; De Smet et al., 2010). This LRP arrest is bypassed when the *slr CYCD3;1^OE^* line is treated with exogenous auxin. The treatment re-establishes normal LRP morphogenesis, which is accompanied by restoration of *ARABIDOPSIS CRINKLY4* (*ACR4*) and *PLT3* expression, which are downregulated in *slr- CYCD3;1^OE^* (De Smet et al., 2010). Therefore, activation of cell proliferation is necessary but not sufficient to trigger LRP morphogenesis, and both correct auxin signaling and cell proliferation are required for normal LRP morphogenesis (Figure 5). It seems that auxin is linked to cell proliferation in this context through an F-box protein S-Phase Kinase-Associated Protein 2A (SKP2A) (Jurado et al., 2010).

**FIGURE 6:**
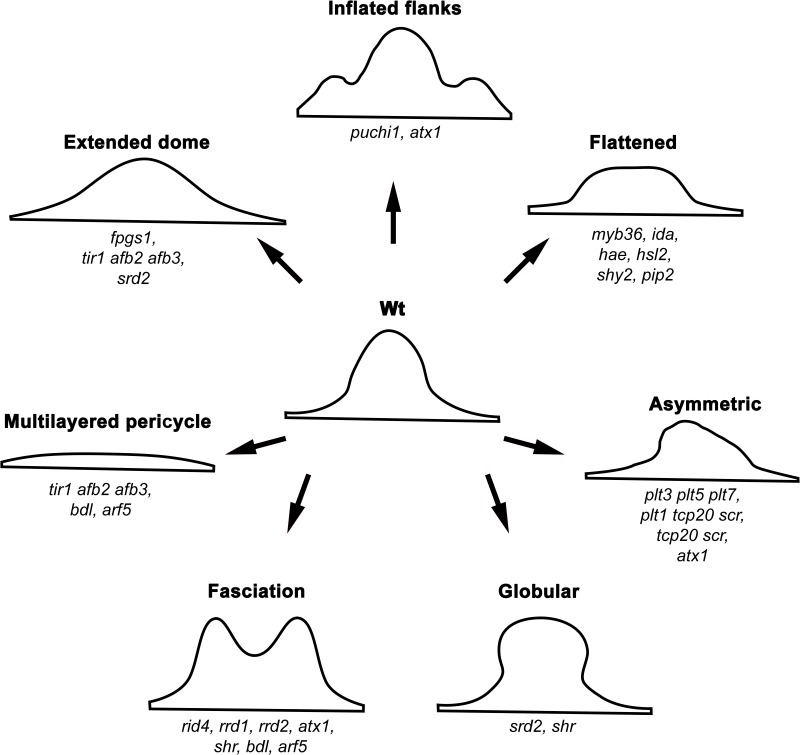
Categories of lateral root primordium phenotype abnormalities in different *Arabidopsis* mutants. See text for details.

Another important auxin-signaling module involved in LRP morphogenesis was revealed based on phenotypic analysis of the *bodenlos (bdl)/iaa12* and *monopteros/arf5* mutants (De Smet et al., 2010). In these mutants, a multilayered pericycle and fused LRPs show clear abnormalities in morphogenesis (Figure 6), demonstrating again that auxin signaling is of paramount importance for organized LRP development. Similarly, in the triple mutant affected in proteins involved in auxin perception, TRANSPORT INHIBITOR RESPONSE1 (TIR1), AUXIN SIGNALING F-BOX2 (AFB2), and AFB3 (Dharmasiri et al., 2005; Kepinski and Leyser, 2005), LRP morphogenesis turns out to be abnormal, resulting either in a multilayered pericycle or unusually wide LRPs (Figure 6) (Dubrovsky et al., 2011).

Cytokinins (CKs) are likewise important for LRP morphogenesis. CK biosynthesis takes place in the developing LRPs as shown using the PHOSPHATES-ISOPENTENYL TRANSFERASE *pIPT5::GFP* reporter (Takei et al., 2004). CKs are negative regulators of LR development (Li et al., 2006; Laplaze et al., 2007; Marhavý et al., 2011; Bielach et al., 2012). Specifically, CKs inhibit LRP initiation or some of the anticlinal divisions in StI LRPs (Li et al., 2006; Laplaze et al., 2007; Bielach et al., 2012). Later stages of LRP development are also inhibited, but they are less sensitive to CKs and, for this reason, time from initiation to emergence is increased (Li et al., 2006; Laplaze et al., 2007; Bielach et al., 2012). CK-treated plants show abnormal patterns of cell division throughout LRP morphogenesis. During the early stages, a series of periclinal divisions in external and internal layers occurs out of sequence (Laplaze et al., 2007). These irregularities at the cellular level result in a flattened LRP (Laplaze et al., 2007).

Cytokinins affect the auxin response maximum in developing LRPs, diffusing or abolishing it (Laplaze et al., 2007; Marhavý et al., 2011) through downregulation of PIN expression (Laplaze et al., 2007). From StIII onwards, low concentrations of exogenous N6-benzyladenine (a synthetic cytokinin) promote depletion of PIN1 in the plasma membrane to a greater extent at anticlinal cell walls than at periclinal ones (Marhavý et al., 2014). In this manner, cytokinin modulates the polarity of PIN1, allowing auxin to flow toward the LRP central apical domain (Figure 2) (Marhavý et al., 2014). In support of this notion, in Wt, PIN1 is predominantly found at periclinal cell walls, while in CK receptor mutants, PIN1 becomes localized at anticlinal walls (Marhavý et al., 2014). This CK-dependent redistribution of PIN1 in the mutants does not permit maintenance of the same number of cells in the external layer of the StIV LRP (Marhavý et al., 2014). This analysis clearly demonstrates that both auxins and CKs are involved in maintaining LRP morphogenesis and that crosstalk between these hormones is essential at all LRP developmental stages.

The role of other hormones in LRP morphogenesis is unclear. Ethylene promotes LR emergence through its effect on cell proliferation, at least in the outer LRP layers (Ivanchenko et al., 2008), but the mechanism has not been addressed. Abscisic acid (ABA) inhibits the emergence of LRPs and promotes its dormancy (De Smet et al., 2006b; Fukaki and Tasaka, 2009). In the parent root, a fraction of arrested or slowly developing LRPs is frequently found (Dubrovsky et al., 2006; Napsucialy-Mendivil and Dubrovsky, 2018). The most plausible scenario is that ABA inhibits cell proliferation in the developing LRP, keeping it ‘dormant,’ but this is yet to be shown.

Knowledge of the role of brassinosteroids and gibberellins (GA) in LRP morphogenesis is fragmentary (Fukaki and Tasaka, 2009). In poplar (*Populus* sp.), GA negatively regulates LRP initiation (Gou et al., 2010), but its role in LRP formation is unknown. The *Gibberellic Acid Stimulated-Like* (*GAST-like*) gene family, regulated by GA, is suggested to be involved in LRP development in rice (*Oryza sativa*) and *Z. mays*, but its exact role has yet to be established (Zimmermann et al., 2010). Nitric oxide was recently considered to be a phytohormone (Santner and Estelle, 2009). It inhibits LRP initiation but does not affect LR emergence (Lira-Ruan et al., 2013); however, its role in LRP morphogenesis is unknown. Many—if not all—of the hormone signaling pathways converge at one point or another. These interactions can potentially influence various aspects of LRP morphogenesis and further investigation is needed to address this complex cross-talk.

## Mechanical Forces and Lateral Root Emergence

Even from early studies, it was known that developing LRPs experience mechanical stress imposed by the overlaying tissues (Pond, 1908). Additionally, the LRP is influenced by external factors such as substrate particles, soil compaction, and parent root curvatures. Roots in soil frequently meet mechanical barriers. The internal and external mechanical forces affect LRP morphogenesis, and we will briefly review what is known in this respect.

When roots were grown in beds of glass spheres, roots curved and LR initiation occurred on the external (convex) root side (Goss, 1977; Goss and Russell, 1980). Moreover, when a root is bent during the gravitropic response, or after permanent or transient manual bending that can be as short as 20 s, the LR is also formed on the convex root side (Ditengou et al., 2008; Laskowski et al., 2008; Lucas et al., 2008a,b; Richter et al., 2009; Kircher and Schopfer, 2016). At the bending site, auxin induces AUX1 expression within the root stele of the young differentiation zone, which together with PIN protein reorientation promotes increased auxin transport toward the convex side of the root, creating a positive feedback loop that results in greater auxin levels and eventually in LR initiation (Ditengou et al., 2008; Laskowski et al., 2008).

The possibility of inducing LR initiation at the desired time and place provides a useful experimental system for studying LRP morphogenesis. With this approach, it has been shown that shootward (basipetal) transport of solutes is important for LR initiation and LRP morphogenesis. After root apical meristem removal and experimental root bending (Ditengou et al., 2008) or only after bending (Lucas et al., 2008b), the LRP develops faster on the convex side compared to intact roots. Interestingly, manual root bending promotes LRP initiation in *slr/iaa14* and *arf7 arf19* mutants and they become capable of proceeding with LRP morphogenesis, though in the latter mutant only LRPs but not LRs are formed (Ditengou et al., 2008). Similarly, the ability of other auxin-related mutants to form LRPs and proceed with morphogenesis increases significantly after root bending (Richter et al., 2009). Specifically, these mutants are those affected in *AUX1*, *AUXIN RESISTANT4 (AXR4)* encoding an accessory protein involved in correct localization of AUX1 (Dharmasiri et al., 2006), and *TRANSPORT INHIBITOR RESPONSE1 (TIR1)* encoding the F-box protein auxin receptor (Tan et al., 2007). Overall, these experiments show a clear link between root bending, LR initiation, auxin accumulation at the convex root side, and the rate of LRP formation. The reasons for accelerated LRP formation in bent roots have not yet been addressed. A potential increase in auxin content during LR initiation could impact posterior development of the LRP. An increase of cytosolic Ca^2+^ in the pericycle on the convex side of bent root (Richter et al., 2009) could also impact LRP morphogenesis. Finally, mechanical forces affect cell wall properties in the root portion from which the LRP is emerging.

Cell wall remodeling and cell separation in tissues overlying the LRP are well documented and required for LRP protrusion. These processes depend on auxin signaling. Cell wall remodeling enzymes encoded by *PECTATE LYASE1* (*PLA1*) and *PLA2* are active during LRP protrusion (Laskowski et al., 2006). During this process, expression of *EXPANSIN14* (*EXPA14*) (Lee et al., 2013) and *EXPA17* (Lee and Kim, 2013), which encode cell wall remodeling proteins, is activated by LBD18 transcription factor in response to auxin. A loss-of-function mutant *lbd18* is significantly affected in the progression of LRP morphogenesis, resulting in delayed LR emergence (Lee and Kim, 2013; Lee et al., 2013). This demonstrates an auxin signaling-dependent crosstalk in the tissues overlying the developing LRP. A similar delayed LR emergence phenotype is found in the loss-of-function auxin influx carrier mutant *lax3* (Swarup et al., 2008). *LAX3* is expressed in the cortex and epidermis overlying the LRP and is involved in regulating the expression of *AUXIN INDUCED IN ROOT3* (*AIR3*), encoding a subtilisin-like protease (Neuteboom et al., 1999). Furthermore, POLYGALACTURONASES (PG) and a XYLOGLUCAN:XYLOGLUCOSYL TRANSFERASE 6 (XTR6) were reported to be regulated by a signaling pathway mediated by ARF7, ARF19-IAA14 and LAX3 and by the peptide INFLORESCENCE DEFICIENT IN ABSCISSION (IDA) through the leucine-rich repeat receptor-like kinases HAESA (HAE) and HAESA-LIKE2 (HSL2) (Swarup et al., 2008; Kumpf et al., 2013). Consequently, mutations in *IDA*, *HAE*, and *HSL2* result in both delayed LR emergence and flattened LRPs (Kumpf et al., 2013).

A more severe phenotype of no LR emergence and flattened LRPs is found in auxin-treated plants carrying the stabilized variant of *SHY2/IAA3* expressed from the endodermis-specific promoter of *CASPARIAN STRIP DOMAIN PROTEIN* (*CASP1*) (Vermeer et al., 2014). In these transgenic lines, endodermal cells overlying the LRP were unable to decrease their turgor pressure and consequently the cell volume to permit LRP protrusion. The authors also showed that SHY2 activity in the endodermis and not in the cortex and epidermis is important for LR emergence (Vermeer et al., 2014). Furthermore, the aquaporin water channel PLASMA MEMBRANE INTRINSIC PROTEINS (PIPs) have essential roles in the turgor pressure control of both the LRP and the overlying cells (Péret et al., 2012a). As expected, in *pip2* mutants, LRPs are flattened and LRs emerge at a slower rate (Péret et al., 2012a).

These data collectively demonstrate the importance of auxin signaling in the control of cell wall remodeling and turgor pressure in the overlying tissues that impact LRP morphogenesis and LR emergence. However, the mechanism by which the mechanical forces are perceived, the respective signal transduction pathways, the modes of communication between overlying tissues and LRP cells, and the morphogenesis mechanisms dependent on these factors, are still to be discovered.

## Cell Type Identity Acquisition

In vascular plants, three main tissue systems form: dermal, ground, and vascular tissues, originating from the protoderm, periblem, and plerome histogens, respectively (Hanstein, 1870; Esau, 1977; Evert, 2006). The idea that cell types acquire their identity in the very early stages of LRP formation was first deduced from an anatomical retrospective analysis. Going back from advanced LRP stages, when cell types can be recognized, to earlier stages, Von Guttenberg (1960) proposed that the first periclinal division in StI primordium cells forms two layers, the internal and external layer, with different developmental fates. These layers are marked on Figure 1 as IL and OL following Malamy and Benfey (1997). The internal layer is already specified at this early stage as plerome, giving rise to the future vascular cylinder or stele. Next, the external layer of StII LRPs (OL) undergoes a second periclinal division that gives rise to two outer layers (the external OL1 and the internal outer OL2), again with different cell fates. The protoderm (prospective epidermis) and the root cap are specified from OL1 layer, and OL2 gives rise to the periblem (prospective ground tissue; Von Guttenberg, 1960). Thus, the main cell types can be recognized already in StIII LRPs. It seems that this holds true in different taxa (Von Guttenberg, 1960; Voronkina, 1978).

Remarkably, although this conclusion was based on anatomical studies, it was later confirmed using cell type-specific marker lines of *Arabidopsis* (Malamy and Benfey, 1997). Figure 1 illustrates the sequence of the cell type identity acquisitions in the developing LRP based on cell type reporters. It was reported that the enhancer trap markers for different cell types are expressed similarly in the primary and lateral roots. The stele-specific marker *SHORT-ROOT* (*SHR*) is transcribed in the stele of the primary root (Helariutta, 2000), and also in the internal cell layer of the StII LRP (IL), confirming its vascular (stele) identity (Tian et al., 2014; Goh et al., 2016; Du and Scheres, 2017b). The endodermis-specific *SCARECROW* (*SCR*; Di Laurenzio et al., 1996) is expressed in the external layer of the StII LRP (OL), confirming its endodermal identity (Tian et al., 2014; Goh et al., 2016; Du and Scheres, 2017b). After the second periclinal division in OL of StII LRP, both resulting outer layers (OL1 and OL2) maintain endodermal identity, as confirmed by *pSCR::GFP:SCR* expression (Goh et al., 2016). When the third periclinal division occurrs in the StIII LRP, it takes place in the innermost layer (IL). As evidenced by *pSHR::SHR:GFP* expression, both new layers maintain the stele identity (Goh et al., 2016). At this stage (StIV), the endormal cell indentity becomes restricted to the second outer layer (OL2).

When the internal layer of the StIII LRP (IL) divides periclinally, it forms the most internal (IL1) layer, presumably giving rise to pericycle, and the second layer (IL2), giving rise to other provascular tissues of the LRP (Malamy and Benfey, 1997). However, no pericycle-specific markers have been shown to be expressed within the central domains of an LRP. At stages V and VI, the external cells of the central domain of the LRP acquire root cap and epidermis identities, as evidenced by the promoter activity of the NAC domain transcription factor FEZ (Willemsen et al., 2008; Du and Scheres, 2017b) and of the MYB-related transcription factor WEREWOLF (Lee and Schiefelbein, 1999; Du and Scheres, 2017b), respectively. Therefore, the use of cell type-specific marker lines substantially enhanced our understanding of LRP morphogenesis and revealed that practically all meristematic cell type identitites are acquired before LR emergence (Malamy and Benfey, 1997; Du and Scheres, 2017b). These studies clearly demonstrate that differential gene expression involved in cell type identity acquisition starts very early in LRP morphogenesis.

A GFP reporter of the enhancer trap line J0121 is expressed specifically in the protoxylem-adjacent pericycle of the parent root. J0121 GFP expression was detected in all layers of the developing LRP from StI to StIII (Laplaze et al., 2005; Dubrovsky et al., 2006). This suggests that the early LRP cells posess a mixed identity. In the J0121 line, GFP is not expressed in the root apical meristem, but it is expressed throughout the elongation and differentiation zones of the parent root. Importantly, starting from StIV, GFP expression in the J0121 line is excluded from the central domains of the LRP and is maintained in the flanking domains until LR emergence, suggesting that the central and flanking domains have different developmental fates. Furthermore, this pattern suggests that, starting from StIV, the central LRP domain acquires features of a root apical meristem, as it no longer expresses J0121 GFP. This observation is in line with the fact that, from StIV onwards, the LRP becomes less dependent on the parent root (Laskowski et al., 1995), which could be related to the beginning of autonomous auxin synthesis in the LRP. Additionally, a gene regulatory network analysis showed that the formation of the central and flanking domains is controlled by two distinct gene clusters (Lavenus et al., 2015).

As discussed, the first periclinal divisions are developmentally asymmetric (Scheres and Benfey, 1999), i.e., different developmental fates are aquired by the daughter cells. Indeed, each cell type origin is dependent on these asymmetric divisions and we still do not understand how these are regulated. We do not yet know how early pericycle, xylem, phloem, and vascular parenchyma cell type identities are acquired during LRP morphogenesis. Cell type identity acquisition studies with the aid of cell type reporters are limited to *Arabidopsis.* Extension to other angiosperms is needed.

## New Stem Cell Niche Establishment

The QC in roots is a population of slowly cycling cells that gives rise to all the cells of the apical meristem and serves as an organizing center with stem cell properties (Clowes, 1954, 1975; Barlow, 1997; Bennett and Scheres, 2010; Dubrovsky and Barlow, 2015). In some species, such as *Malva sylvestris* (Byrne, 1973) and *V. faba* (MacLeod and McLachlan, 1974; MacLeod, 1977), the QC is established after LR emergence. In other species, such as *Eichornia*, *Pistia* (Clowes, 1958), and *Arabidopsis* (Goh et al., 2016), the QC is established during LRP morphogenesis. In yet other species, e.g., *Z. mays* (Clowes, 1978b), the QC can be established either before or after LR emergence. The establishment of a functional QC requires a critical mass of proliferating cells within a developing LRP. In *Z. mays*, cell proliferation in the CLS of endodermal origin is essential for QC establishment (Clowes, 1978b). This early-established QC vanishes when the CLS cells are sloughed off. Soon after, the new root cap initial cells of pericyclic origin are produced and simultaneously a new cap and QC are established (Clowes, 1978b).

In *Arabidopsis*, the *WUSCHEL-RELATED HOMEOBOX 5* (*WOX5*) gene is specifically expressed in the QC (Sarkar et al., 2007). The p*WOX5*::*GFP* reporter is expressed already at StI (Ditengou et al., 2008), when LRP initiation is triggered by a primary root bending, or at StII in intact roots (Tian et al., 2014; Du and Scheres, 2017b; Shimotohno et al., 2018). This expression pattern, which resembles the expression of *WOX5* soon after the onset of embryogenesis (Sarkar et al., 2007), suggests that the cell lineage that will lead to QC establishment is specified early in root development. Moreover, the possibility exists that correct *WOX5* expression is required and sufficient to define the central domain of the LRP and could provide a hallmark for the neigboring domains (Figure 2). Another QC marker, QC25 (Sabatini et al., 2003; ten Hove et al., 2010), starts to be expressed in the OL2 of StIV LRPs, when the cycle time increases by 70% in that layer compared to that in the most external layer, OL1 (Goh et al., 2016). QC establishment depends on *SCR*, as in the *scr* loss-of-function mutant, the specification of QC identity—as monitored by the *pWOX5::n3GFP* reporter—does not take place in the OL2 LRP layer but in the more internal layers that have stele identity (Goh et al., 2016). QC identity is completely lost in the *plt3 plt5 plt7* mutant, underlying the importance of these transcription factors in QC establishment (Du and Scheres, 2017b). PLT1 or PLT3 and SCR form a protein complex mediated by TCP20/21 (a plant-specific Teosinte-branched-Cycloidea PCNA [Proliferating cell nuclear antigen]) transcription factor that binds to the *WOX5* promoter, and this complex is important for QC specification and LRP morphogenesis (Shimotohno et al., 2018). Triple mutants in genes encoding members of this complex show LRP morphogenesis abnormalities in both the central and flanking domains (Shimotohno et al., 2018). This phenotype reveals a tight link between cell type identity acquisition and morphogenetic processes. The observation that the QC is established before LR emergence suggests that LRP morphogenesis culminates with a new root apical meristem that becomes functional post-emergence. An open question is how the stem cell identiy is acquired during LRP morphogenesis and what other factors are involved in this process. Again, our knowledge of this process for species other than *Arabidopsis* is limited.

## Genetic Control of Lateral Root Primordium Morphogenesis

A morphogenetic process must be considered from a 3D perspective. How gene regulatory networks define an organized structure is a central question for understanding morphogenesis. The 3D structure during embryogenesis was first acquired and fixed in evolution starting from gametophore development in bryophytes. In this basal land plant, rotation in the orientation of the cell division plane in stem cells permitted 3D morphogenesis (Harrison, 2017), an evolutionary novelty involving the *CLAVATA* (*CLV*) signaling pathway (Whitewoods et al., 2018). Time-lapse (4D) analyses of the genetic control of LRP morphogenesis are only beginning to be possible (Lucas et al., 2013; Vermeer et al., 2014; Goh et al., 2016; von Wangenheim et al., 2016) and few detailed studies of mutants affected in LRP morphogenesis have been performed. To examine the genetic control of LRP morphogenesis (Figure 5), we consider mutants that exhibit abnormal LRP formation and attempt to discern which morphogenetic processes are affected.

Some of the first mutants reported to be affected in LRP morphogenesis were *shoot redifferentiation defective (srd2)*, *root initiation defective (rid4)*, and *root redifferentiation* (*rrd1* and *rrd2*) (Yasutani et al., 1994; Konishi and Sugiyama, 2003; Sugiyama, 2003). These mutants were isolated in a temperature-dependent mutant screen aimed at identifying genes involved in root development that could be essential, and thus conditionally lethal if absent, during early developmental stages. In the *srd2* mutant, time from LRP initiation to LR emergence is significantly increased (Ohtani and Sugiyama, 2005; Ohtani et al., 2010). Furthermore, from StV onwards, LRP morphogenesis becomes abnormal. The altered cell division pattern results in the formation of an LRP with a wider dome than in the Wt (Ohtani et al., 2010) due to increased length and thickness of the flanking domains and a decrease in the apical–basal axis length (see the respective domains in Figure 2). This *srd2* phenotype is related to the lack of auxin maximum establishment in the apical domain of the LRP in the mutant, which results from downregulation of the PIN proteins (Ohtani et al., 2010). *SRD2* encodes a nuclear protein that shares sequence similarity with human SOLUBLE NSF ATTACHMENT PROTEIN 50 (SNAP50), a subunit of the SNAPc multiprotein complex required for small nuclear RNA transcription (Ohtani and Sugiyama, 2005; Ohtani et al., 2010). The precise mechanism of *SRD2* action in LRP morphogenesis is unknown.

The phenotype of the *rrd1*, *rrd2*, and *rid4* mutants during the LRP pre-emergence stages is similar to that described for *srd2*. Surprisingly, the post-emergence LR phenotype in these mutants is different from that of *srd2*. While in *srd2*, the LRs are globular without pronounced growth, those of the *rrd1*, *rrd2*, and *rid4* mutants do grow, but produce fasciated (fused) roots (Konishi and Sugiyama, 2003; Sugiyama, 2003). During abnormal LRP development, the internal LRP cells form the prospective stele, which appears as a fusion of two adjacent LRPs. This was shown using *pSHR:GFP* and *pPIN1:PIN1-GFP* reporters. At the same time, *pSCR:GFP* expression revealed that the single external layer corresponding to the developing endodermis encloses the fused steles of the developing LRP (Otsuka and Sugiyama, 2012). The molecular function of genes affected in these mutants remains to be identified. The abnormal cell division pattern, which is more evident when seedlings are transferred from permissive to restrictive temperatures during the earlier stages of LRP morphogenesis, is a common feature of the mutants (Otsuka and Sugiyama, 2012). Furthermore, in the non-temperature-dependent *atx1* mutant affected in a H3K4-methyltransferase, similar LRP phenotypes (wider LRP and fasciated primordia) were found (Napsucialy-Mendivil et al., 2014). Abnormalities in cell division pattern and proliferation in the LRP at different developmental stages are also reported in the *shr* (Lucas et al., 2011) and *folylpolyglutamate synthetase1* (*fpgs1*) mutants (Reyes-Hernández et al., 2014). LRP morphogenesis in the *srd2*, *rrd1*, *rrd2*, *rid4, atx1, shr*, and *fpgs1* mutants shows that correct cell division patterns and proliferation are critical factors in sustaining normal LRP development. In most of these cases, the abnormalities are equally distributed throughout all the LRP domains.

Lateral root primordium morphogenesis could also be affected either at specific stages or in specific LRP domains. Thus, in the *puchi* mutant, anticlinal and periclinal divisions are supernumerary in both the central and flanking domains, resulting in an increased thickness of the flanking domain and an overall flatter LRP at later stages of development (Hirota et al., 2007). *PUCHI* encodes an AP2/EREBP transcription factor that acts downstream of auxin signaling and is most strongly expressed in the flanking and basal domains (Hirota et al., 2007). Thus, *PUCHI* is apparently required to limit the extent of cell proliferation in the flanking domain and to restrict it to the central domain during LRP morphogenesis (Hirota et al., 2007). Similarly, *myb36* mutant LRPs are flatter than those of the Wt and, from StIV onwards, a defect in the transition from the flat to dome-shaped LRP is observed (Fernández-Marcos et al., 2017). More cells along the central basal and flanking domains of the *myb36* LRP are produced, resulting in wider LRPs than in the Wt (Fernández-Marcos et al., 2017). MYB36 is a transcription factor expressed in the LRP from StV onwards and is restricted to the central basal and flanking domains, where it controls the expression of peroxidases PER9 and PER64 (Fernández-Marcos et al., 2017). Thus, MYB36 apparently regulates LRP width through limiting cell proliferation mediated by changes in reactive oxygen species (ROS) balance.

In addition to the genetic control of morphogenesis at specific times and places, another aspect of morphogenesis is the control of cell division patterns and the orientation of the cell division plate. In the *aurora* (*aur*)*1 aur2* double mutant, oblique or irregularly shaped divisions take place during StI, after the first 2–3 anticlinal divisions. Therefore, the typical layered structure of the LRP is not formed (Van Damme et al., 2011). *AUR1* and *AUR2* encode Ser/Thr kinases that phosphorylate Ser 10 of Histone H3 during mitosis (Demidov et al., 2005, 2009; Kawabe et al., 2005). A recent *in vitro* study showed that AUR1 interacts and phosphorylates the SHR transcription factor; however, this interaction has not been confirmed *in planta* (Takagi et al., 2016). Interestingly, despite altered orientation of cell division throughout LRP development, the overall shape of the primordium is not significantly affected (Lucas et al., 2013). Nevertheless, LR emergence is substantially delayed in the *aur1 aur2* mutant (Van Damme et al., 2011; Lucas et al., 2013).

Other genes controlling the cell division orientation are those encoding PLT transcription factors. The triple mutant *plt3 plt5 plt7* is characterized by multiple defects in LRP formation, including irregular cell shapes, aberrant LRP morphology, and a lack of layered LRP structure (Hofhuis et al., 2013; Du and Scheres, 2017b). *PLT1, PLT2*, and *PLT4* are expressed at later stages during LRP development and their expression depends on the expression of *PLT3, PLT5*, and *PLT7*, which are expressed from StI onward. Therefore, the *plt3 plt5 plt7* mutant is affected in these six *PLT* genes, highlighting their importance in LRP morphogenesis. An overview of the genetic control of LRP morphogenesis is presented in Figure 5, while categories of the outlined abnormal LRP phenotypes are shown in Figure 6.

Together with genetic analysis, transcriptomic approaches (Brady et al., 2007; Moreno-Risueno et al., 2010) have also been used recently to analyze the genetic control of LRP morphogenesis. Together, these two approaches permit construction of gene regulatory networks and thus contribute to the identification of genes involved in LR development (Lavenus et al., 2015; Voß et al., 2015). Recent papers report how the architecture of gene regulatory networks changes during LRP formation. In one such study, the gravistimulation-induced LRP system (Lucas et al., 2008a) was implemented and time-series expression data sets collected starting from LRP initiation. In this way, two mutually exclusive gene clusters regulated by auxin were identified that act in non-overlapping central and flanking domains of the LRP (Lavenus et al., 2015). One cluster involves regulation by ARF7 and ARF19 and first acts in both domains, but soon becomes restricted to the flanking domain, where it is maintained until LR emergence. The second cluster is regulated by MP, ARF6, and ARF8 and acts in the central LRP domain (Lavenus et al., 2015). This analysis confirmed the importance of previously known genes involved in LRP morphogenesis and allowed the identification of new gene regulatory network nodes that potentially participate in LRP morphogenesis (Lavenus et al., 2015). Experimental validation of the identified genes will settle their particular roles in this process.

A transcriptomic approach was also used to identify new genes involved in LRP formation. For this, cell sorting of roots expressing the *pSKP2B:GFP* reporter, which is active at all LRP stages (Manzano et al., 2012), was performed and used to identify SKP2B-coexpressed genes (Manzano et al., 2014). This analysis revealed genes involved in ROS signaling, among others. One of these genes, *UPBEAT1* (*UPB1*), encodes a bHLH transcription factor and is expressed in the flanking LRP domain (Manzano et al., 2014). UPB1 regulates the expression of a subset of *PEROXIDASE* (*PER*) genes involved in maintaining the ROS balance (Tsukagoshi et al., 2010; Manzano et al., 2014). LR emergence is significantly delayed in *per7* and *per57* loss-of-function mutants and is promoted in the *PER7* overexpression line, suggesting the importance of ROS in LRP morphogenesis through UPB1-mediated signaling (Manzano et al., 2014). In line with these studies, RESPIRATORY BURST OXIDASE HOMOLOGS (RBOH) NADPH oxidases that produce extracellular ROS are also involved in LRP development (Manzano et al., 2014; Orman-Ligeza et al., 2016). Interestingly, nitroblue tetrazolium (NBT) staining, employed for the localization of superoxide, was detected in the central domain, but staining was absent or much lower in the flanking LRP domains (Manzano et al., 2014). In double and triple *rboh* mutants, LR emergence was also delayed, while it was accelerated in *RBOHD*-overexpressor lines (Orman-Ligeza et al., 2016). Collectively, these studies demonstrate that ROS promote progression of LRP formation and that redox state is important for LRP morphogenesis, even though it is not known which specific morphogenetic processes are involved. Overall, transcriptomic approaches permit efficient identification of many new players involved in LRP formation and further studies should clarify their roles.

## Lateral Root Morphogenesis and Plant Health

A better understanding of LRP morphogenesis is important for both basic and applied science. Correct root primordium morphogenesis is the foundation of a healthy root system and appropriate root architecture. Abnormal primordium morphogenesis is a characteristic of rhizomania disease of sugar beet (*Beta vulgaris*) (D’ambra et al., 1972; Pollini and Giunchedi, 1989). This disease is induced by the beet necrotic yellow vein virus (BNYVV) and causes supernumerary LR formation on the taproot, leading to a dramatic decrease of root mass and yield. The viral P25 virulence factor mimics auxin action by deregulating BvAUX28. As a result, some root-specific LBD transcription factors and EXPANSINS are upregulated, which in turn promote uncontrolled LR formation (Gil et al., 2018) and probably cause abnormal morphogenesis. Early processes of LR development affected by this and other root diseases are underexplored, and studies of these diseases may suggest strategies to control and/or prevent abnormalities in LRP morphogenesis.

## Concluding Remarks and Future Perspectives

Here we outlined the main components of LRP morphogenesis in angiosperms. Each facet of LRP morphogenesis reflected in the respective sections of this review outlines specific open questions. Most data on the genetic control of LRP morphogenesis are available for *Arabidopsis* and can be used for comparative studies in angiosperms. Further understanding of LRP morphogenesis in crop species is needed to modulate or adjust root system architecture to specific growth conditions.

The main tendencies, important for further research in this field, are related to the development of new technologies that could be used to address the open questions. These tendencies are as follows:

(1) Addressing the genetic control of morphogenesis in mutants and Wt plants by 3D analysis in time (4D) can significantly advance our understanding of root system formation. New imaging technologies and new microscopy approaches (Ovečka et al., 2018) that could be used for this purpose are already accessible. Deciphering cell division patterns and developmental rules involved in morphogenesis (Yoshida et al., 2014; von Wangenheim et al., 2016) is an important goal.
(2) Gene regulatory networks uncover complex relationships between pathways involved in regulating different processes during LRP morphogenesis (Lavenus et al., 2015; Voß et al., 2015). Further studies of gene regulatory networks at the single cell level and implementation of plant systems biology approaches (Libault et al., 2017) will undoubtedly contribute to answering the questions of how different LRP domains and cell types are specified and maintained, how the overall shape of the developing LRP emerges, and how timing control is operated.
(3) LRPs develop under mechanical constraints imposed by the external parent root tissues. The role of mechanical forces during LRP morphogenesis was recognized in early studies, but only recently did their roles in both LR initiation (Vermeer et al., 2014) and morphogenesis (Stoeckle et al., 2018) begin to be understood. Models of auxin transport coupled to mechanical forces provide explanations for the robust morphogenesis observed in the *Arabidopsis* root (Romero-Arias et al., 2017) and application of these models to LRP morphogenesis should be promising. Developing new biophysical methods to monitor the mechanical properties of live cells (e.g., Elsayad et al., 2016) is challenging, but required to discern the role of the mechanical forces in LRP morphogenesis. LRP formation is closely linked to external and internal mechanical forces and the cytoskeleton (Eng and Sampathkumar, 2018), but it is unclear how the mechanical forces contribute to LRP morphogenesis.
(4) We outlined a possible role of the temporal CLS formed during LRP morphogenesis and attempted to visualize related evolutionary trends of this particular feature of LRP morphogenesis. Understanding the relationships between different facets of LRP morphogenesis (e.g., orientation of cell division, developmental rules, participation of different cell types) and how they change during evolution is challenging but feasible in the post-genomic era. Integration of different approaches from genomics and molecular to cell biology and anatomy could help reveal evo–devo relationships in LRP morphogenesis of angiosperms.

Here we reviewed the main aspects of LRP morphogenesis that have been under investigation for more than a century. Not all available information was discussed; for instance, we did not include the role of environmental factors and mineral nutrition. We hope that the historical perspective combined here with our overview of contemporary studies of LRP morphogenesis highlights key questions that will guide future research aimed at elucidating the morphogenetic processes that take place during LRP development. Such research would yield important insights into root biology and evolution, providing a framework to modulate root system architecture, root production and root adaptation to the environment in crop species.

## Author Contributions

HT-M and JD conceived the idea and designed the outlines of this review article. SS and JD conceptualized the content. HT-M and JD wrote the article. GR-A performed phylogenetic and gene regulatory network analyses. GR-A, HT-M, and JD prepared the illustrations. All authors participated in the editorial improvement of the text and approved the final manuscript.

## Conflict of Interest Statement

The authors declare that the research was conducted in the absence of any commercial or financial relationships that could be construed as a potential conflict of interest.
